# Influence of Plasma Jet Temperature Profiles in Arc Discharge Methods of Carbon Nanotubes Synthesis

**DOI:** 10.3390/nano7030050

**Published:** 2017-02-23

**Authors:** Grzegorz Raniszewski, Slawomir Wiak, Lukasz Pietrzak, Lukasz Szymanski, Zbigniew Kolacinski

**Affiliations:** Institute of Mechatronics and Information Systems, Lodz University of Technology, 90-924 Lodz, Poland; slawomir.wiak@p.lodz.pl (S.W.); lukasz.pietrzak@p.lodz.pl (L.P.); lukasz.szymanski@p.lodz.pl (L.S.); zbigniew.kolacinski@p.lodz.pl (Z.K.)

**Keywords:** carbon nanotubes, arc discharge, plasma jet, temperature measurement

## Abstract

One of the most common methods of carbon nanotubes (CNTs) synthesis is application of an electric-arc plasma. However, the final product in the form of cathode deposit is composed of carbon nanotubes and a variety of carbon impurities. An assay of carbon nanotubes produced in arc discharge systems available on the market shows that commercial cathode deposits contain about 10% CNTs. Given that the quality of the final product depends on carbon–plasma jet parameters, it is possible to increase the yield of the synthesis by plasma jet control. Most of the carbon nanotubes are multiwall carbon nanotubes (MWCNTs). It was observed that the addition of catalysts significantly changes the plasma composition, effective ionization potential, the arc channel conductance, and in effect temperature of the arc and carbon elements flux. This paper focuses on the influence of metal components on plasma-jet forming containing carbon nanotubes cathode deposit. The plasma jet temperature control system is presented.

## 1. Introduction

Carbon nanotubes (CNTs) are new materials with unique properties discovered in 1991 [1]. Nowadays, CNTs are used in almost every area of life. There are many methods of carbon nanotubes synthesis. The most common low-temperature technique is chemical vapor deposition (CVD) and the best-described high-temperature methods are arc discharge methods and laser ablation [2,3,4,5,6,7,8,9,10,11,12]. In our research, we focus on arc methods. Although this method used nowadays is well known and is recognized in waste treatment, technology, medicine and microbiology [13,14,15,16], the basic processes involved in nanotechnology and deposit formation are still under investigation. In the CVD methods, relatively high temperature (hundreds of K) decomposes gases containing carbon [17]. In the arc method, the temperature of the arc (thousands of K) leads to vaporization and decomposition of the graphite electrode. Additional elements such as Fe, Ni, Co, Y, S, Cr, etc. can be used as catalysts to obtain CNTs with unique properties [18,19,20,21,22,23,24,25,26,27]. There are a variety of parameters which influence the plasma stream such as plasma composition, distance between electrodes, pressure, current, and voltage. All parameters directly or indirectly influence the plasma column conductance, resistance and in effect—temperature [28]. One of the methods for plasma column parameters measurement is a temperature measurement based on the ratio of two spectral line intensities [29].

Due to their electrical, optical, chemical and physical properties, carbon nanotubes are widely used in almost every industry. They have been on the market for years and, every month, hundreds of articles about CNTs are published. Despite this, the cost of pure nanotubes—especially functionalized ones—may exceed 100 USD/g. One way to change this situation is improvement of the synthesis efficiency. To increase the yield, we must improve control of the synthesis process, especially the essential parameter, which is the temperature.

Metal particles (catalysts) in the plasma column strongly change its parameters such as thermal conductivity, effective ionization potential and temperature. Stability of the plasma, and in effect stability of the temperature distribution, determines the quality of the final product. So, an effective monitoring and control system is required for the synthesis process optimization. It may be realized by plasma jet temperature profile measurement.

## 2. Temperature Measurement

### 2.1. Arc Discharge Set-Up

**Figure 1:**
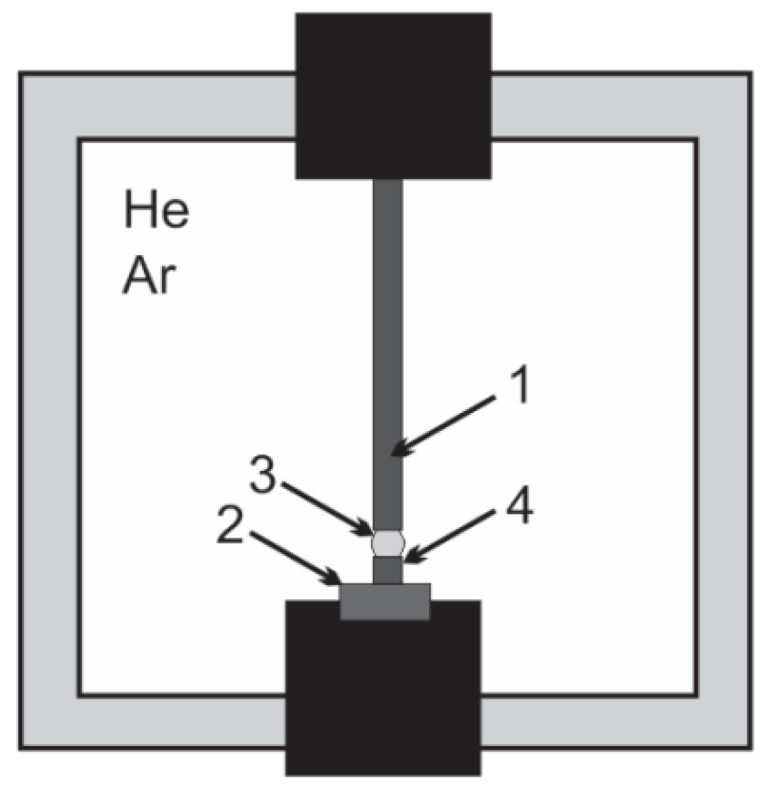
Arc discharge carbon nanotubes synthesis system where: 1—anode; 2—cathode; 3—vapour jet; 4—cathode deposit.

In the arc discharge methods, the synthesis process occurs in a low-pressure neutral atmosphere. In this case, graphite carbon electrodes are used as a source of carbon (Figure 1). The distance between electrodes does not exceed 1 mm. A temperature of the arc discharge near the anode surface is higher than the boiling point of carbon. This temperature causes vaporization of the carbon elements and leads to formation of plasma jet. Carbon elements form a carbon–plasma jet and are deposited on the relatively cold cathode. The product consists of different carbon molecules: soot, amorphous carbon, fullerenes, and multiwall carbon nanotubes.

Arc discharge methods enable synthesis of multiwall carbon nanotubes. It is necessary to apply catalysts to the synthesis of single-wall carbon nanotubes or to the synthesis of the carbon nanotubes with special properties e.g., ferromagnetic. The most effective catalyst elements are from ferrous group metals [30,31,32,33]. The anode may be filled with a catalyst or carbon–catalyst mixture. During vaporization, metal particles with carbon particles go towards the cathode-forming metal–carbon plasma column. In our research, we focused on metals such as Ni, Co, Y and Fe. The carbon nanotube growth occurs in the environment composed of ions of gas (e.g., He, Ar), ions of carbon, multi-atom molecules, neutral gas particles, ions of catalyst, neutral catalyst particles and electrons. As an essential parameter, the profile of plasma stream temperature is chosen. It was found that morphology of the product depends on the coalescence of carbon individuals and catalysts in the colder reaction zone (out of the plasma). In the case of electric-arc discharges, even a small amount of metal in plasma increases electron density and electrical conductivity, resulting in a temperature increase [34].

The synthesis process begins with the repetitive evacuation of the research chamber and filling up by ambient gas in order to eliminate oxygen. Then, it is filled with argon, helium or a mixture of these gasses with the pressure between 0.2 bar and 0.5 bar. The positioning system moves the anode leading to the connection of the two electrodes. Then the current flowing through the electrodes heats them up, which enables an electric-arc ignition.

**Figure 2:**
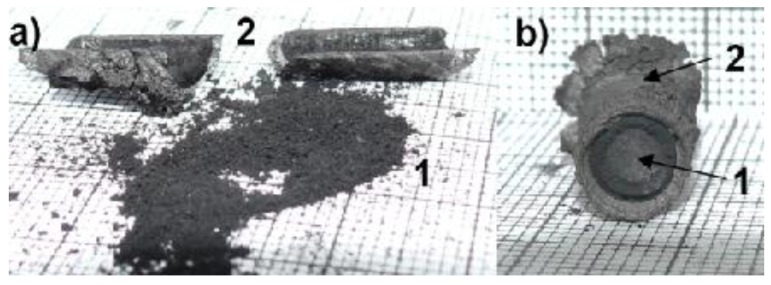
Cathode deposit where 1—soft core with nanotubes; 2—hard shell; (**a**) separated parts of deposit; (**b**) deposit cross-section.

After a few seconds, the space between the electrodes is increased and the automatic control system keeps the distance between the electrodes during the whole process. The high temperature of the arc leads to vaporization of the anode. Carbon or carbon-metal vapors—depending on a catalytic or non-catalytic process—go along the arc channel and decompose on the cathode surface, forming the irregular cylindrical cathode deposit (Figure 2).

**Figure 3:**
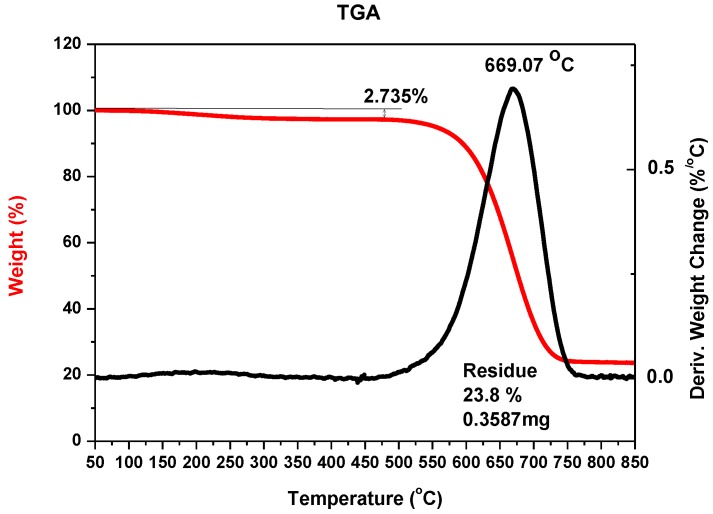
Thermogravimetric analysis (TGA) of the sample with carbon nanotubes with iron nanoparticles after oxidation (example for 5% *w*/*w* iron as catalyst, pressure 200 hPa, current 70 A, voltage 22.5 V).

**Figure 4:**
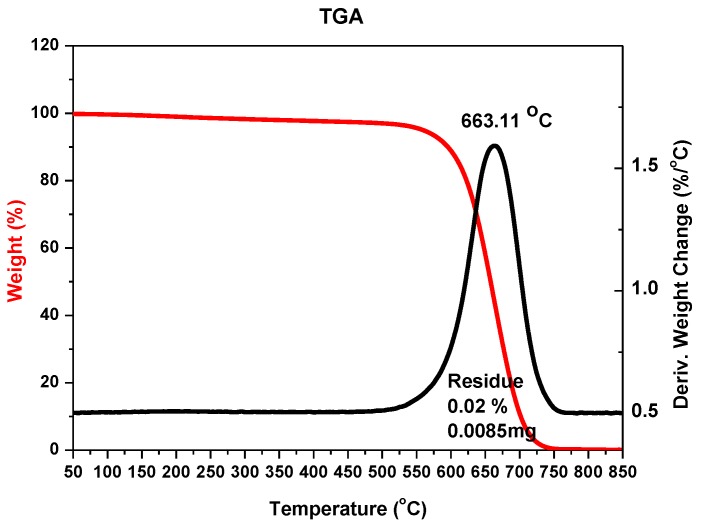
TGA analysis of the sample with carbon nanotubes after oxidation (pressure 200 hPa, current 80 A, voltage 21 V).

A temperature control system of the plasma jet during the synthesis process makes it possible to obtain deposits with a maximum soft core/hard shell ratio. Application of too high temperatures leads to thermal destruction of nanotubes. In too low temperatures, deposit is not formed. Cathode deposit consists of two parts: carbon hard shell and soft core. Soft core is composed of different forms of carbon: graphite, amorphous carbon and nanotubes. These two parts can easily be mechanically separated from each other. Soft core may contain at least 30% *w*/*w* nanotubes, depending on the plasma jet temperature. The final product is a mixture of components and, for industry use, usually requires additional purification. One of the simplest methods of purification is a physical method called “dry oxidation”. This kind of oxidation is carried out in air, oxygen or ozone, usually at an elevated temperature. It is assumed that heating of carbon materials at temperatures 720–750 K in air causes oxidation of soot, fullerenes and nanotubes with defects and sometimes spherical ends of nanotubes. More resistant are carbon nanotubes (630–675 K). At temperatures above 870 K, destruction of not-deformed multiwall carbon nanotubes is completed. To evaluate the purity of the obtained product, thermogravimetric analysis (TGA) and scanning electron microscopy (SEM) were applied. For thermogravimetric analysis, 2950 TGA HR, TA Instruments was used. For microscopic analysis, JEOL JSM 5500 LV was used. Figure 3 shows an example of TGA analysis for carbon nanotubes synthesized with iron as catalyst. Figure 4 shows an example of analysis for carbon nanotubes obtained without catalyst. 

It can be noticed that the largest mass reduction appeared at the temperature of about 950 K, which suggests the presence of large amounts of carbon nanotubes in the sample, as multiwalled carbon nanotubes decomposition temperature occurs within 850–950 K [35]. The residue—23.76% indicates the amount of metal nanoparticles in the sample. 

**Figure 5:**
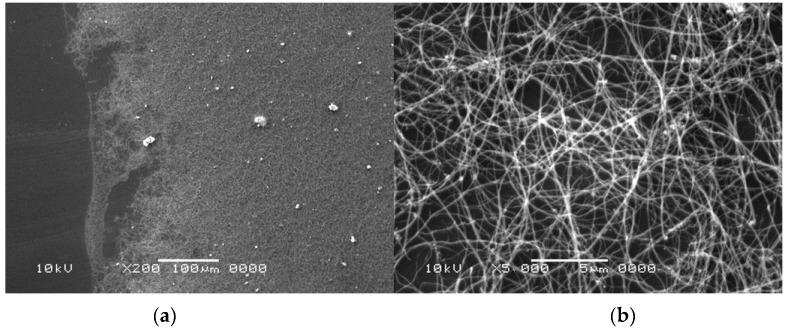
Scanning electron microscopy (SEM) analysis of the sample with carbon nanotubes after oxidation (pressure 200 hPa, current 80 A, voltage 21 V). Figures shows different magnification of the sample—100× on (**a**); 5000× on (**b**).

Figure 5 shows an exemplary result of SEM analysis—dispersed multiwall carbon nanotubes (MWCNTs) on Si wafer. The left side of the figure shows a typical SEM image of MWCNTs’ “spaghetti” and part of the Si wafer surface. Brighter objects are catalyst particles. The right part shows an image of MWCNTs in higher magnification.

### 2.2. The Plasma Jet

Although in the applied systems we use under pressure it is assumed that the plasma column is in the near-local thermodynamic equilibrium state [36], due to a high evaporation ratio, the local pressure between two electrodes is relatively high. The assumption that plasma is in the local thermodynamic equilibrium state means that:

- temperatures—the average kinetic energy of particles—of all of the plasma components are equal;
- velocity distribution of all kinds of particles is described in Maxwell’s law;
- distribution of particles with different energy levels is defined by the Boltzmann law;
- the plasma components concentration is defined by the Saha–Eggert equation;
- concentration of the individual components of chemical reactions is determined by the Guldberg–Waage law of mass action.

**Figure 6:**
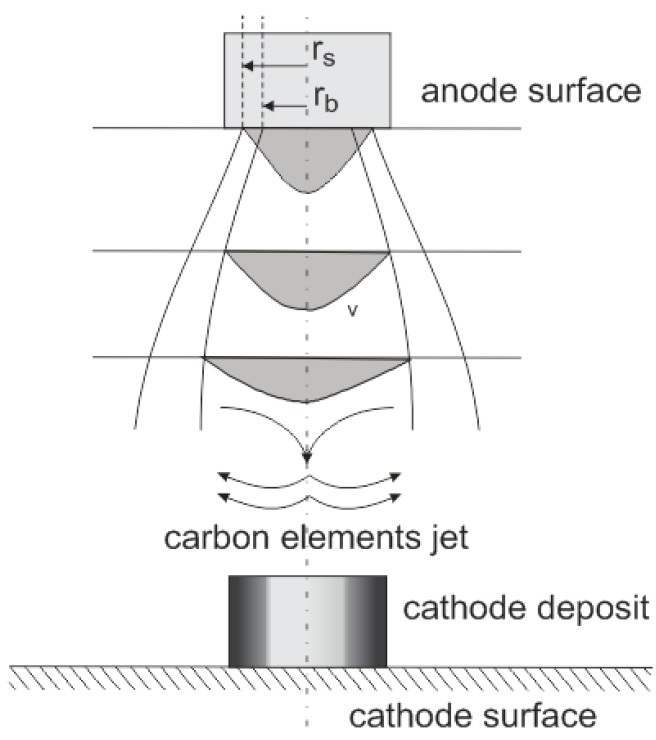
Anode vapor jet generation where: *r*_s_—sublimation radius; *r*_b_—boiling radius; *v*—velocity of carbon profile.

Due to the small distance between electrodes, the diameter of the radius of the plasma column is relatively high. Cathode deposit growth depends on the mass of carbon elements transported from the anode to the cathode. The average velocity distribution of carbon vapor over the anode spot surface can be assumed as Gaussian (Figure 6).

**Figure 7:**
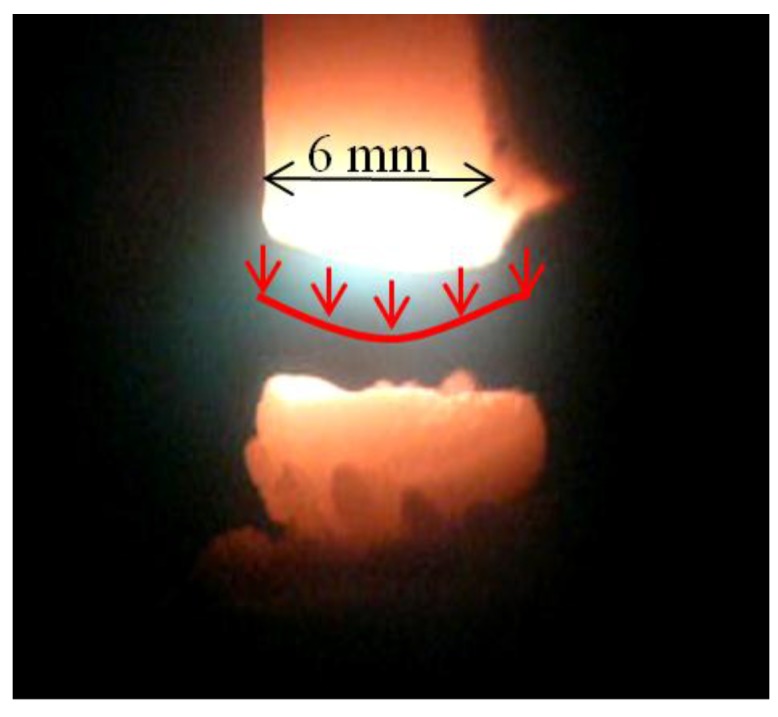
Plasma column gap in arc plasma carbon nanotubes (CNTs) synthesis.

The effectively evaporating area of the anode spot is bound with boiling radius *r*_b_, but the arc channel foot is based on the sublimation radius *r*_s_. The temperature profile is also similar to Gaussian and equals 4500–5500 K in the arc axis [37,38]. Figure 7 shows the photo of the plasma jet with schematically indicated carbon elements velocity flux.

### 2.3. Temperature Measurement

One of the methods for plasma temperature measurement is the method whereby two spectral lines characteristic for the same element are used. In this method, the image of the plasma column goes to a spectrograph. The plasma temperature can be evaluated from the expression based on the spectral lines characteristic for the same element [39,40,41].

Light from the plasma column reaches the optical system. Either moving parts of the optical system or matrix of sensors can record the full profile of the arc. After reaching the spectroscope, light and its change over time are recorded and analyzed. By selecting the two spectral lines belonging to the same element coming from the catalyst, it is possible to record intensity changes over time. Spectral lines used for calculation should have the following characteristics:

- a relatively high intensity;
- spontaneous transition probabilities and statistical weights should be well described in literature;
- density of spectral lines in the separated region should be small.

**Figure 8:**
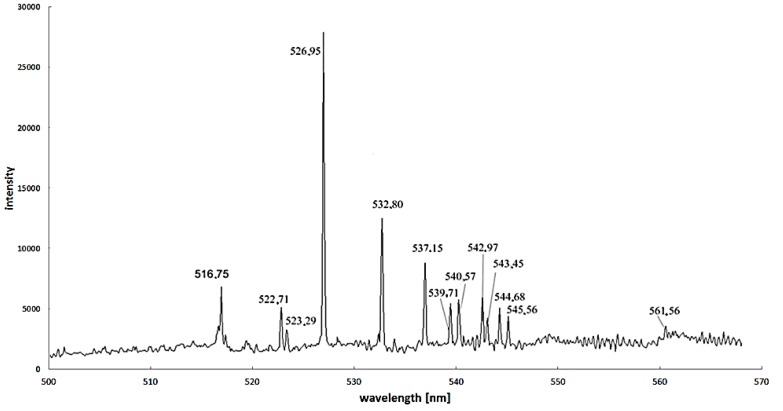
Spectra for arc discharge in the discharge axis during carbon nanotubes synthesis supported by iron as a catalyst (recorded by Stellarnet HR-NIR4, Stellarnet Inc., Tampa, FL, USA, spectrometer).

Excitation energy difference is also an important factor to increase the calculation accuracy. For iron—as the most popular catalyst—the proper spectral lines are 445.91 nm and 446.17 nm, but spectral lines 495.76 nm, 522.71 nm and 526.95 nm can also be used (Figure 8) [37].

It was calculated that even a small amount (up to 1%–3%) of catalysts is sufficient to detect as a specific spectral line in all spectrums [42]. Knowledge of the intensity ratio allows for determining the temperature:

(1) $$T=\;\frac{\chi_{l}-\;\chi_{n}}{\ln\left(\frac{A_{lk}\cdot g_{l}}{A_{nm}\cdot g_{n}}\right)-\ln\left(\frac{\lambda_{2}}{\lambda_{2}}\right)-\ln\left(\frac{\varepsilon_{v_{1}}}{\varepsilon_{v_{2}}}\right)}$$

where: χ—excitation energy, respectively for levels *l* and *n*, *A*—spontaneous transition probability, *g*—statistical weight, λ—spectral lines wavelengths, ε—the intensity of the spectral lines calculated by using the Abel transformation of the measured values.

**Figure 9:**
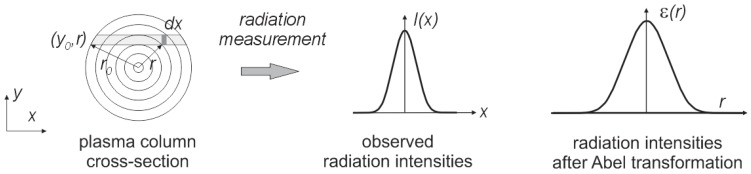
The observed distribution of radiation along the arc cross-section.

It was assumed that the synthesis of carbon nanotubes forming the plasma column is circular and is axially symmetrical over its entire length. Perpendicular measurement of plasma radiation is a sum of the radiation through the thickness of the arc column. To calculate the intensity of radiation at a given point in the column, Abel transformation should be used [37]. The plasma can be divided into *n* concentric spaced rings (Figure 9).

By Abel transformation we can obtain:

(2) $$\varepsilon \left(r\right)=-\frac{1}{\pi }\overset{r}{\underset{x}{\int }}\frac{dI\left(x\right)}{dx}\cdot \frac{dx}{\sqrt{x^{2}-r^{2}}}$$

To calculate the temperature, the Nestor and Olsen [43] method has been used:

(3) $$\varepsilon_{\lambda }\left(k\right)=\frac{2}{\pi \cdot \delta_{x}}\overset{n}{\underset{i=k}{\sum }}B_{k,i}I_{\lambda ,\;i}$$

where:

(4) $$B_{k,i}=\frac{1}{2k+1}$$

for *i* = *k*,

(5) $$B_{k,i}=\frac{\sqrt{i^{2}-k^{2}}-\sqrt{{\left(i-1\right)}^{2}-k^{2}}}{2i-1}-\frac{\sqrt{{\left(i+1\right)}^{2}-k^{2}}-\sqrt{i^{2}-k^{2}}}{2i+1}$$

for *i* ≥ *k* + 1.

This method requires good recognition of the atomic data of the plasma column-creating elements. Most of the catalysts used in arc discharge plasma are well described in literature [44]. Additionally, the measured spectral lines should have a relatively large intensity compared to the background radiation. The use of spectral lines in areas with a large concentration of them can cause accumulation of the number of lines and affect the results of the readings.

## 3. Results

**Figure 10:**
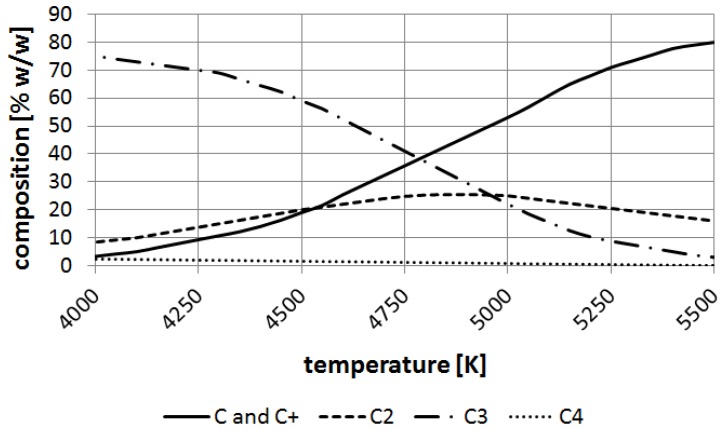
Decomposition of carbon at temperatures 4000–5500 K.

The ChemSage software (GTT Technologies, Herzogenrath, Germany) has been applied for calculations of carbon element equilibrium. It can be noticed that carbon particles such as carbon ions, C_2_, C_3_ appear over 4000 K and at the boiling point of carbon (about 4800 K) C and are balanced nearly to 30% (*w*/*w*) (Figure 10). 

**Figure 11:**
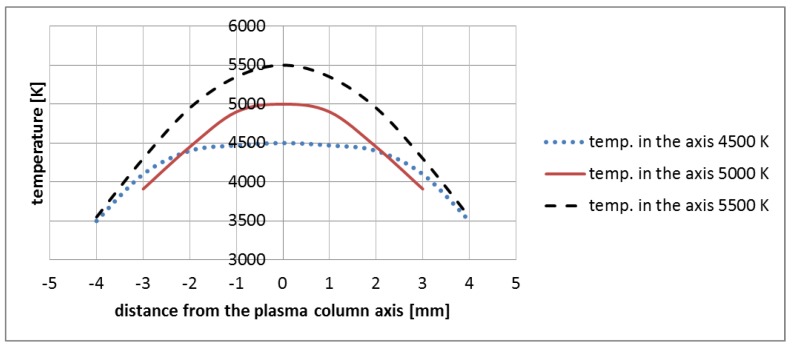
Examples of temperature profiles with average temperature in the axis: 4500 K, 5000 K and 5500 K (for the distance between electrodes −2 mm; pressure 200 hPa; helium as an inert gas; current 70 A, 80 A, 90 A respectively).

Figure 11 shows examples of measured temperature profiles for average temperatures in the axis 4500 K, 5000 K and 5500 K. This temperature depends on current, distance between electrodes, and the plasma composition. 

**Figure 12:**
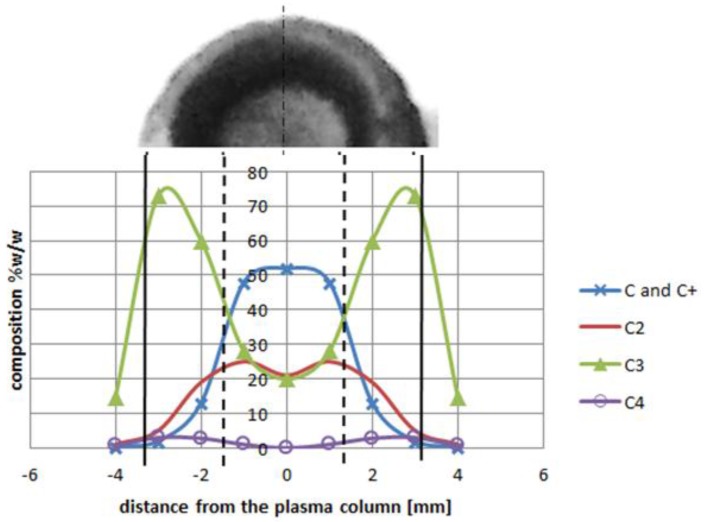
Plasma column composition with carbon deposit structure (**top**). Deposit obtained under conditions: pressure 300 hPa, current 80 A, voltage 20.5 V.

**Figure 13:**
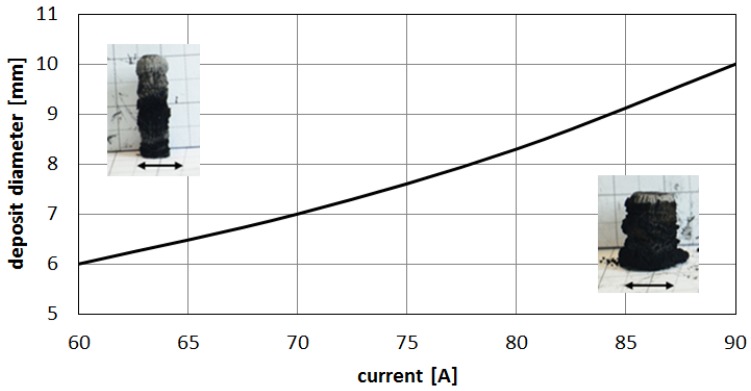
Influence of the discharge current on deposit size.

At temperature 5000 K, vapor jet of C and C_2_ is sufficient for fulfilling the arc discharge region with carbon elements. The helium—or helium and argon—elements can be omitted due to a high carbon/gas ratio in the plasma arc zone. The carbon ions dominate in the plasma stream as the particles are heated up to a temperature of 5500 K. If we compare decomposition of carbon in a temperature range characteristic for the arc profile, it is possible to obtain profiles of carbon elements in the plasma jet (Figure 12). A strong influence of the temperature on plasma composition, and then on the size and structure of the deposit, can be seen (Figure 13). 

**Figure 14:**
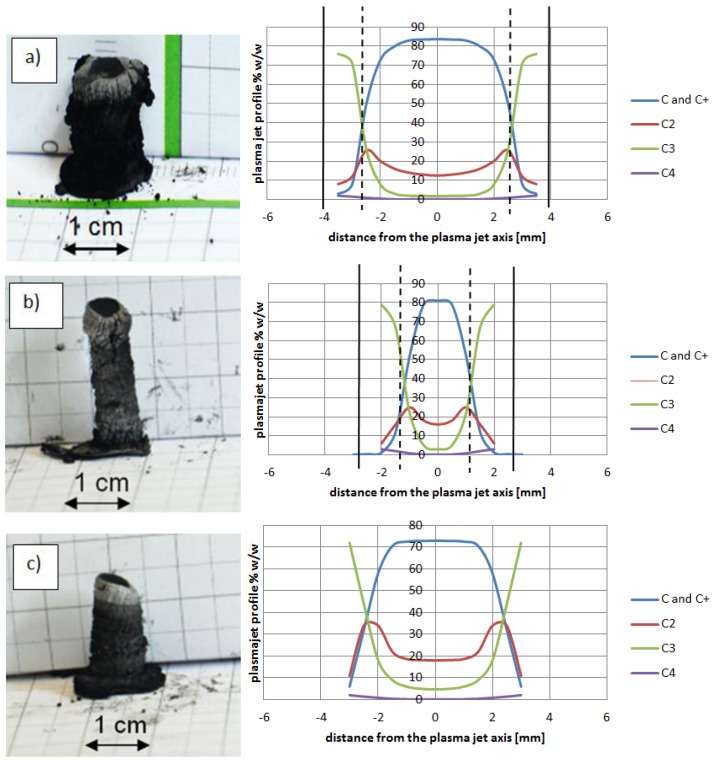
Examples of cathode deposits (photos in the left and plasma column composition in the right) where (**a**) Ni as catalyst (5 wt %, pressure 200 hPa, current 90 A, voltage 26 V); (**b**) Y as catalyst (5 wt %, pressure 200 hPa, current 60 A, voltage 25 V); (**c**) Fe as catalyst (5 wt %, pressure 200 hPa, current 70 A, voltage 22.5 V).

Higher arc current increases anode jet velocity. Intensifying evaporation results in an increase in carbon vapor partial pressure near the cathode deposit surface. The growth rate can be controlled by the combination of arc current and the gap between electrodes. It can be noticed that containing carbon nanotubes soft core corresponds to the predominance of small carbon elements and carbon ions near the discharge axis. Multiatom carbon compounds appear in the zone of hard-shell formation. Figure 14 shows composition of plasma jet for different catalysts introduced into the system.

The plasma stream temperature measurement, and then the recalculation temperature into plasma jet composition, explains the differences between cathode deposits morphology. Control of the plasma column temperature—and thus plasma composition—during synthesis enlarges the volume of soft core, which leads to synthesis improvement. 

## 4. Summary

It was noticed that the minimum metal content in the plasma arc determines the essential properties of the plasma. The addition of catalysts changes the plasma composition, electrons density, conductance and in effect—temperature of the plasma. This fact affects the carbon nanotubes synthesis efficiency.

The calculation of the temperature is independent on concentrations of elements and plasma composition, but choice of the proper lines is essential. Spectral lines should have a relatively high intensity, spontaneous transition probabilities and statistical weights should be well described in literature, density of spectral lines in the separated region should be small. However, the difference in excitation energies (χ_1_ − χ_2_) is also an important factor that can increase the calculation accuracy.

For iron as the most popular catalyst, the proper couple of spectral lines are 445.91 nm and 446.17 nm, but spectral lines 495.76 nm, 522.71 nm and 526.95 nm can also be used; for Ni lines with relatively high intensity: 499.2 nm and 503.54 nm; for Co: 453.1 nm and 456.56 nm. 

The big advantage of this method is that temperature calculation is independent on concentration of plasma elements and plasma composition does not affect the measurement accuracy. The disadvantage is that accuracy depends on the spectral lines chosen.

Knowledge about carbon decomposition in different temperatures with the information about the temperature profile enables us to determine the profile of carbon elements stream and then the structure of the deposit.

The results of experiments and calculations have great practical potential in modelling of the phenomena that occurs during the synthesis process. A new approach to the problem will improve currently used technologies and increase the efficiency of carbon nanotube creation. Plasma temperature is one of the most important factors during carbon nanotubes synthesis by arc discharge method. However, this temperature is the consequence of the combined effect of the pressure, the type of ambient gas, the type of applied catalysts, the catalyst contamination, the method for introducing the catalyst into the system, the arc current, the distance between electrodes and even the system configuration. The combination of numerous parameters makes the process almost unpredictable and forces empirical determination of the best conditions for carbon nanotubes synthesis. Determining the plasma jet temperature simplifies the control system. It was measured that by controlling the plasma jet temperature profile, it is possible to increase the carbon nanotubes efficiency by up to 30%. In our arc discharge system, where the plasma jet diameter equals about 6–10 mm, the most appropriate temperature is the temperature over 4800 K in the center part of the plasma, and lower temperature at the edges. The lower temperature is responsible for the creation of larger particles of carbon, which form hard-shell closing carbon nanotubes inside deposit. It was observed that the most appropriate situation is when the internal higher temperature is included in 60–80% of the plasma jet.
